# *CHST11* gene expression and DNA methylation in breast cancer

**DOI:** 10.3892/ijo.2015.2828

**Published:** 2015-01-09

**Authors:** DAMIR HERMAN, TATIANA I. LEAKEY, ALICE BEHRENS, AIWEI YAO-BORENGASSER, CRAIG A. COONEY, FARIBA JOUSHEGHANY, BOUNLEUT PHANAVANH, ERIC R. SIEGEL, A. MAZIN SAFAR, SOHEILA KOROURIAN, THOMAS KIEBER-EMMONS, BEHJATOLAH MONZAVI-KARBASSI

**Affiliations:** 1 Division of Hematology/Oncology, University of Arkansas for Medical Sciences, Little Rock, AR 72205, USA; 2 Department of Pathology, University of Arkansas for Medical Sciences, Little Rock, AR 72205, USA; 3 Department of Medical Genetics, University of Arkansas for Medical Sciences, Little Rock, AR 72205, USA; 4 Department of Biochemistry and Molecular Biology, University of Arkansas for Medical Sciences, Little Rock, AR 72205, USA; 5 Department of Biostatistics, University of Arkansas for Medical Sciences, Little Rock, AR 72205, USA; 6 Winthrop P. Rockefeller Cancer Institute, University of Arkansas for Medical Sciences, Little Rock, AR 72205, USA; 7 Research and Development, Central Arkansas Veterans Healthcare System, Little Rock, AR 72205, USA

**Keywords:** breast cancer, chondroitin sulfate, CHST11, gene expression, DNA methylation, 5-aza-2′-deoxycytidine

## Abstract

Our previously published data link P-selectin-reactive chondroitin sulfate structures on the surface of breast cancer cells to metastatic behavior of cells. We have shown that a particular sulfation pattern mediated by the expression of carbohydrate (chondroitin 4) sulfotransferase-11 (*CHST11*) correlates with P-selectin binding and aggressiveness of human breast cancer cell lines. The present study was performed to evaluate the prognostic value of *CHST11* expression and determine whether aberrant DNA methylation controls *CHST11* expression in breast cancer. Publicly available datasets were used to examine the association of *CHST11* expression to aggressiveness and progression of breast cancer. Methylation status was analyzed using bisulfite genomic sequencing. 5-aza-2′-deoxycytidine (5AzadC) was used for DNA demethylation. Reduced representation bisulfite sequencing was performed in the CpG island of *CHST11* with a minimum coverage of 10. Quantitative real-time RT-PCR was employed to confirm the expression profile of *CHST11* in breast cancer cell lines. Flow cytometry was also used to confirm the expression of the CHST11 product, chondroitin sulfate A (CS-A). The expression of *CHST11* was significantly higher in basal-like and Her2-amplified cell lines compared to luminal cell lines. *CHST11* was also highly expressed in cancer tissues compared to normal tissues and the expression levels were significantly associated with tumor progression. We observed very low levels of DNA methylation in a CpG island of *CHST11* in basal-like cells but very high levels in the same region in luminal cells. Treatment of MCF7 cells, a luminal cell line with very low expression of *CHST11*, with 5AzadC increased the expression of *CHST11* and its immediate product, CS-A, in a dose-dependent manner. These results suggest that *CHST11* may play a direct role in progression of breast cancer and that its expression is controlled by DNA methylation. Therefore, in addition to CHST11 mRNA levels, the methylation status of this gene also has potential as a prognostic biomarker.

## Introduction

Increased expression and alteration in sulfation pattern of chondroitin sulfate glycosaminoglycans (CS-GAGs) are observed in various neoplastic tissues, including pancreatic, lung, ovarian and breast (1–3). Several studies, including ours, suggest involvement of particular CS chains in tumor progression and metastasis (2,4–7). The sulfation by site-specific chondroitin sulfotransferases controls the biological function of CS-GAGs (8–11). CHST11 is a key enzyme in the biosynthesis of chondroitin sulfates (CS) and its action on chondroitin chains can lead to the production of chondroitin sulfate A (CS-A), B (CS-B) and E (CS-E) (12,13). We have shown that the expression of *CHST11* is upregulated in tumor tissues of breast cancer patients compared to their normal tissues and that the expression levels of the gene in breast cancer cell lines correlate with CS-A expression, P-selectin binding and aggressive phenotype (14). However, the clinical significance of CHST11 expression is yet to be established and more studies are needed to evaluate the expression of this gene in relation to cancer progression.

We have observed that the expression of *CHST11* is low to none in MCF7 cells with considerably higher expression in MDA-MB-231 (14). The expression levels correlated with P-selectin reactivity and general aggressiveness. The expression of P-selectin ligands on tumor cells plays a role in distant metastasis by facilitating tumor cell extravasation (15–18). Because of its potential role in breast cancer metastasis, determining mechanisms controlling *CHST11* expression is of great interest. Aberrant DNA methylation is a mechanism that controls gene expression and is involved in tumor initiation and progression (19–23). In a gene profiling study of head and neck cancer it was suggested that DNA methylation may affect *CHST11* expression in laryngeal carcinoma (24). In another study on breast cancer cell lines using genome wide methylation profiling, the authors reported that *CHST11* is hypermethylated in ER-positive and hypomethylated in ER-negative cell lines (25). Therefore, variation in the expression of *CHST11* in breast cancer cells may be controlled by DNA methylation.

Our current data suggest that the expression levels of *CHST11* can predict progression of breast cancer and more aggressive phenotypes. Our investigation of the methylation status of *CHST11* in breast cancer cells clearly demonstrates an association between lack of expression of this gene and hypermethylation of a CpG island of its DNA. Treatment of MCF7 cells with 5AzadC led to a dose-dependent increase in the expression of the gene and its product CS-A. Given the role of CS and the significance of *CHST11* expression in tumor growth and metastasis, these findings have significant implications for the development of novel prognostic strategies in breast cancer.

## Materials and methods

### Reagents

Anti-CS-A mAb 2H6 was from Associates of Cape Cod/Seikagaku America (Falmouth, MA, USA). Fluorescence-conjugated, anti-mouse IgM and 5-aza-2′-deoxycytidine (5AzadC) were from Sigma (St. Louis, MO, USA). Primers were from Integrated DNA Technologies (IDT, Coralville, IA, USA). Real-time PCR reagents were from Applied Biosystems (Foster City, CA, USA). TRIzol reagent was from Invitrogen (Carlsbad, CA, USA), FailSafe PCR PreMix Selection kits and Enzyme Mix were from Epicentre Biotechnologies (Madison, WI, USA). Alkaline phosphatase (rAPid) was from Roche (Nutley, NJ, USA).

### Analysis of Oncomine cancer gene microarray database

Publicly available Oncomine cancer microarray database (Compendia Biosciences; Ann Arbor, MI, USA; www.oncomine.com) was used to examine the expression of *CHST11* in cancer tissue and determine association of *CHST11* expression with breast cancer outcomes. Richardson *et al* (GEO accession GSE3744) (26), Finak *et al* (GEO accession GSE9014) (27), The Cancer Genome Atlas (TCGA, http://tcga-data.nci.nih.gov/tcga/) invasive breast carcinoma gene expression data and Gluck *et al* (GEO accession GSE22358) (28) dataset were used to compare *CHST11* expression levels between cancer and normal tissues. Hoeflich *et al* (GEO accession GSE12777) (29) dataset was used to compare expression levels of *CHST11* among a panel of cell lines that represent luminal, Her-2-amplified and basal-like molecular subtypes of breast cancer. Schuetz *et al* dataset (GEO accession GSE3893) (30) was used to compare *CHST11* expression levels between matched ductal carcinoma *in situ* (DCIS) and invasive ductal carcinoma (IDC). Log-transformed, median-centered and normalized expression values (31) were extracted, analyzed and graphed accordingly.

### Cell lines and tissue specimen

Cell lines MCF7, MDA-MB-231, MDA-MB-468, T47-D, and ZR-75-1 were from ATCC (Manassas, VA, USA). MDA-MB-231, MDA-MB-468, T47-D, and ZR-75-1 cells were cultured in DMEM supplemented with 10% heat-inactivated fetal bovine serum (Life Technologies), 50 U/ml penicillin and 50 μg/ml streptomycin. MCF7 were grown as described before (14). Cells are checked every six months to be free from Mycoplasma contamination using the MycoAlert^®^ Mycoplasma Detection kit (Lonza Rockland Inc., Rockland, ME, USA).

De-identified paraffin-embedded specimens from 5 female breast cancer patients were provided by the Department of Pathology of the University of Arkansas for Medical Sciences (UAMS). For this study, an active human tissue use protocol approved by the UAMS Institutional Review Board was used.

### Total RNA isolation and quantitative real-time RT-PCR (real-time RT-qPCR)

RNA isolation and quantitative real-time was performed as described earlier (14). Briefly, total RNA was isolated from cultured cells using TRIzol reagent (Life Technologies, Grand Island, NY, USA) following the manufacturer’s instructions. The quantity and quality of the isolated RNA was determined using an Agilent 2100 Bioanalyzer (Palo Alto, CA, USA). Total RNA (1 μg) was reverse-transcribed using random-hexamer primers with TaqMan Reverse Transcription reagents (Applied Biosystems). Reverse-transcribed RNA was amplified with SYBR Green PCR Master Mix (Applied Biosystems) plus 0.3 μM of gene-specific upstream and downstream primers during 40 cycles on an Applied Biosystems 7900 HT Fast Real-time system. Data were analyzed by absolute and relative quantification. In absolute quantification, data were expressed in relation to 18S RNA, where the standard curves were generated using pooled RNA from the samples assayed. In relative quantification, the 2^−ΔΔC_T_^ method was used to assess the target transcript in a treatment group to that of untreated control using expression of an internal control (reference gene) to normalize data (32). Expression of GAPDH was used as internal control. The primer sequences are shown in Table I.

### 5AzadC treatment

5AzadC was dissolved in ice-cold phosphate-buffered saline, filter sterilized at 4°C and the resulting solution used to treat the MCF7 cell line. For dose–response experiments, cells were harvested 5 days after the initial treatment. Cell growth medium was refreshed every other day.

### Extraction and bisulfite modification of DNA

DNA from cells was extracted as described before (33). DNA from the paraffin-embedded tissues of breast cancer patients was extracted using Ex-Wax DNA extraction kit (Chemicon International, Temecula, CA, USA), following the manufacturer’s instructions with the additional steps of extracting recovered DNA with phenol (Amresco, Solon, OH, USA) and then 1-bromo-3-chloropropane (Molecular Research Center Inc., Cincinnati, OH, USA). DNA was bisulfite modified with an Epitect kit (Qiagen, Valencia, CA, USA) using 300 ng of DNA per reaction. PCR was performed using a FailSafe PCR PreMix Selection kit and FailSafe Enzyme Mix. Each 25-μl PCR reaction included 1.0 μM of each primer, 2.5 U of the FailSafe Enzyme Mix and 12.5 μl of the FailSafe PCR PreMixes A or C. Bisulfite-modified genomic DNA was amplified by semi-nested PCR using two sets of primers for part of intron 1 that is within a CpG island spanning exon 1 of the CHST11 gene (Genbank NM_000012 and exon 1 located with NM_018413). The same amplification profile was used for both reactions of the semi-nested PCR: 1 cycle at 80°C for 1 min, 1 cycle at 94°C for 1 min; 1 cycle at 95°C for 1 min, 54°C for 1 min, 72°C for 1 min; 1 cycle at 95°C for 1 min, 53°C for 1 min, 72°C for 1 min; 1 cycle at 95°C for 1 min, 52°C for 1 min, 72°C for 1 min; 1 cycle at 95°C for 1 min, 51°C for 1 min, 72°C for 1 min; 36 cycles at 95°C for 1 min, 50°C for 1 min, 72°C for 1 min; 72°C for 5 min and cooling to 4°C.

A forward, outside primer and reverse primer were used for *CHST11* for the first reaction (Table I). A second, semi-nested, PCR was then performed on 1 μl of the amplificate (in a 25-μl PCR reaction) using a forward nested primer and the reverse primer from the first reaction (346-bp PCR product, Table I). The primers were designed using MethPrimer web software (34) (http://www.urogene.org/methprimer/). The CpG island was defined using CpG Island Searcher set on the default criteria for defining CpG islands (35) (http://cpgislands.usc.edu/cpg.aspx).

### Bisulfite genomic sequencing (BGS) and methylation level quantification

BGS was performed as described before (33) with the following minor modifications. We used rAPid alkaline phosphatase and PCR products were sequenced using the nested forward (upstream) *CHST11* primer. We also analyzed DNA methylation in the region spanning >1.5 kb of the *CHST11* CpG island with reduced representation bisulfite sequencing [RRBS (36,37)] data generated on breast cancer cell lines for the Illumina Idea Challenge (http://www.illumina.com/landing/idea) (25). RRBS selects DNA fragments ≤220 bp in the vicinity of MspI recognition sites (C.CGG) (36). We only considered CpG dinucleotides with coverage >10x. The percentage of methylation was inferred from the number of methylated reads divided by the sum of methylated and unmethylated reads. All reads were mapped to the hg18 release of the human genome (http://genome.ucsc.edu/cgi-bin/hgGateway?db=hg18). Second generation sequencing DNA methylation results were plotted in Matlab (http://www.mathworks.com).

### Statistical analysis

One-way ANOVA with Tukey’s *post hoc* procedure test was used to compare gene expression between subtypes of cell lines. For comparison of gene expression data generated by real-time PCR, the raw amount for each mRNA was log transformed and normalized to the control mRNA (18S) amount, and analyzed via one-way ANOVA with Tukey’s *post hoc* procedure. The Mann-Whitney U test was performed to compare gene expression between DCIS and IDC. For 5AzadC induced fold change in gene expression, the mRNA levels of the non-zero doses for each transcript and experimental replication were normalized to that of the zero-dose control, transformed to their base-10 logarithms and analyzed for trend with dose via one-way ANOVA. Statistical analyses were performed using Excel (Microsoft, Seattle, WA, USA) or GraphPad Prism version 5.00 for Windows (GraphPad Software, San Diego, CA, USA). All P-values were 2-sided.

## Results

### CHST11 is overexpressed in basal-like and HER2-amplified cell lines and the elevated expression correlates with tumor progression

We have shown that the expression of *CHST11* is elevated in tumor cells compared to normal cells of breast cancer patient tissue specimens (14). We used Oncomine database to confirm our original data. The data from several studies were analyzed to compare *CHST11* expression in breast carcinoma versus normal breast tissue (Table II). The comparison showed average increases of 2–3.6-fold in *CHST11* expression in breast cancer compared to normal tissue.

**Figure 1:**
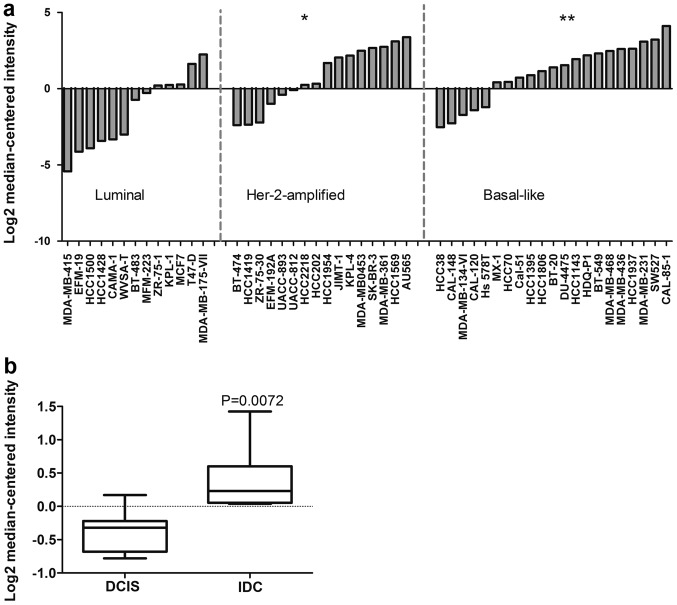
*CHST11* expression is associated with basal-like molecular subtype and progression of breast cancer. All data are from the Oncomine database. (a) *CHST11* gene expression was evaluated in a panel of breast cancer cell lines (13 luminal, 16 Her-2-amplified and 21 basal-like cell lines) (29). The log-transformed normalized expression values were analyzed by one-way ANOVA and Tukey’s *post hoc* comparisons. ^*,**^Statistically significant compared to the luminal subgroup at P<0.05 and P<0.01, respectively. (b) *CHST11* expression is associated with tumor progression (30). Tumor specimens (7 DCIS and 7 IDC) were isolated by LCM from each individual specimen and matched. Specimens then were compared for the expression of *CHST11*. The P-value shows two-sided comparison using Mann-Whitney U test.

Our previously published data analyzing *CHST11* expression in a set of commonly used human breast cancer cell lines suggest that *CHST11* expression is low in luminal and high in basal-like cell lines (14). The data relate *CHST11* expression to basal-like cancer cells. To further investigate such an association, we analyzed an Oncomine dataset that screened 50 breast cancer cell lines representative of the molecular subtypes luminal, Her2-amplified, and basal-like (29). We observed that the expression of *CHST11* in basal-like cell lines was significantly higher than in luminal cell types (Fig. 1a). *CHST11* was also significantly overexpressed in Her-2-amplified cell lines. Considering cells with above average expression as positive, 75% of basal-like and 56% Her-2-amplified cell lines were positive; while only 15% of luminal cell lines were positive for *CHST11* expression.

To investigate the association of *CHST11* overexpression with tumor progression, we interrogated another dataset in the Oncomine database, comparing the expression of *CHST11* between ductal carcinoma *in situ* (DCIS) and invasive ductal carcinoma (IDC) (30). DCIS and IDC samples were isolated by laser capture microdissection (LCM) and matched for each individual specimen (30). We found a significant increase in the expression of *CHST11* in IDC compared to DCIS samples (Fig. 1b). Notably, both groups displayed a similar distribution pattern for ER status (5 ER-positive specimens in each group) and HER2-amplification pattern (3 HER2-amplified specimens in each group) with histological grades 2 and 3. The data link the elevated expression of *CHST11* to aggressive subtypes and progression of the disease.

### A CpG island in the CHST11 gene sequence is hypermethylated in MCF7 and hypomethylated in MDA-MB-231 cells

**Figure 2:**
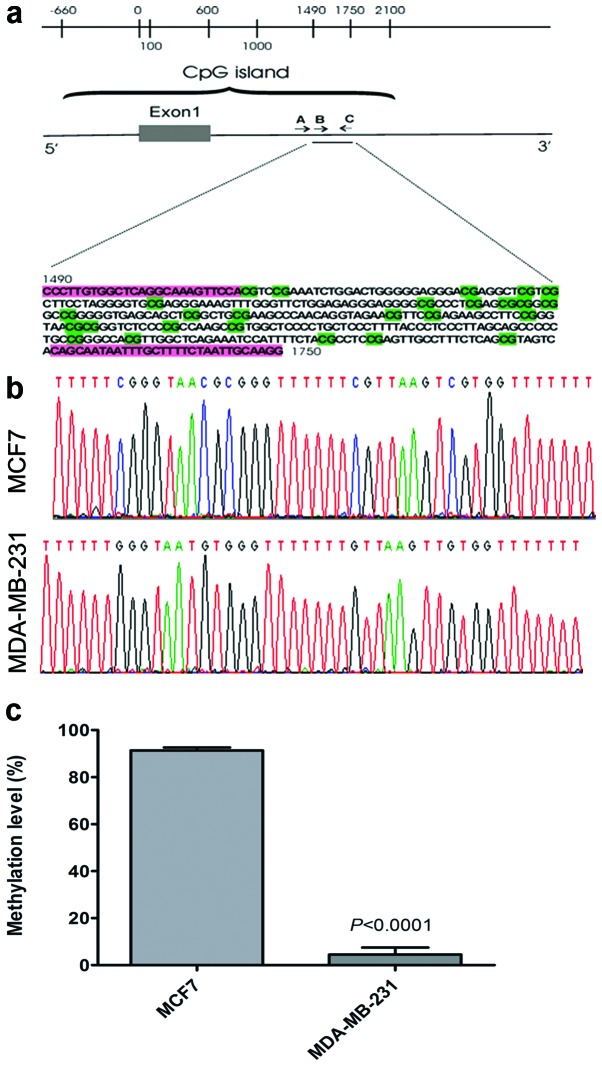
The genomic configuration of the *CHST11* gene and methylation of CPG island in MCF7 and MDA-MB-231 cells. (a) Exon 1 from NCBI NM_018413.5 is shown (0 to +616) as is the CpG island (between −660 and +2,100). The horizontal arrows indicate the approximate relative positions of bisulfite genomic sequencing primers for semi-nested PCR. Primer A is the outside forward primer (Table I), primer B is the nested forward primer (position +1,490 and Table I), and primer C is the reverse primer (position +1,750 and Table I). The genomic sequence (pre-bisulfite) between the B and C primers is shown below the map and B and C primer locations are highlighted in pink. The scale is in base pairs. (b) Sections of bisulfite genomic sequencing electropherograms showing part of the *CHST11* CpG island. Top, DNA of MCF-7 cells. In this top electropherogram most CpG sites have prominent cytosine (C) peaks because methylated cytosines are not changed to thymines (Ts) in the bisulfite reaction. Bottom, DNA of MDA-MB-231 cells. In this section CpG sites appear as TpG sites and are in the same sequence positions as the top CpGs. In this bottom electropherogram most CpG sites have prominent T peaks because unmethylated Cs are changed to uracils in the bisulfite reaction. (c) Quantification of methylation levels of the CpG island in MCF7 and MDA-MB-231 cells. Methylation levels were averaged >10 CpGs and 18 preparations for MCF7 and 16 preparations for MDA-MB-231 cells. Means and SEM are shown. Data were Arcsin transformed and subjected to the Mann-Whitney U test to compare means.

Our current and previously published data suggest that CHST11 expression plays a role in tumor progression and metastasis. Therefore understanding the mechanisms controlling *CHST11* gene expression will help formulate strategies for development of biomarkers and drug targets. We demonstrated previously that *CHST11* and CS-A expression was high in highly metastatic MDA-MB-231 cells, while it was significantly lower in MCF7 cells as assayed by qRT-PCR and flow cytometry (14). To examine whether DNA methylation controls variation and regulation of *CHST11* in breast cancer cell lines, we examined DNA methylation of the *CHST11* sequence in a section of its CpG island covering part of its promoter, its first exon, and a portion of its first intron (Fig. 2a). Using BGS and the Mquant algorithm (33) we determined methylation levels of ten CpG sites for MCF7 and MDA-MB-231 cells (Fig. 2b). This revealed very high methylation levels (91%) averaged >10 CpGs in the *CHST11* sequence in MCF7 cells, and very low levels (5%) over the same 10 CpGs in MDA-MB-231 cells (Fig. 2c). These observations suggest that low expression of *CHST11* in MCF7 cells is due to DNA hypermethylation.

### Treatment of MCF7 cells with 5AzadC increases the expression of CHST11 and CS-A

**Figure 3:**
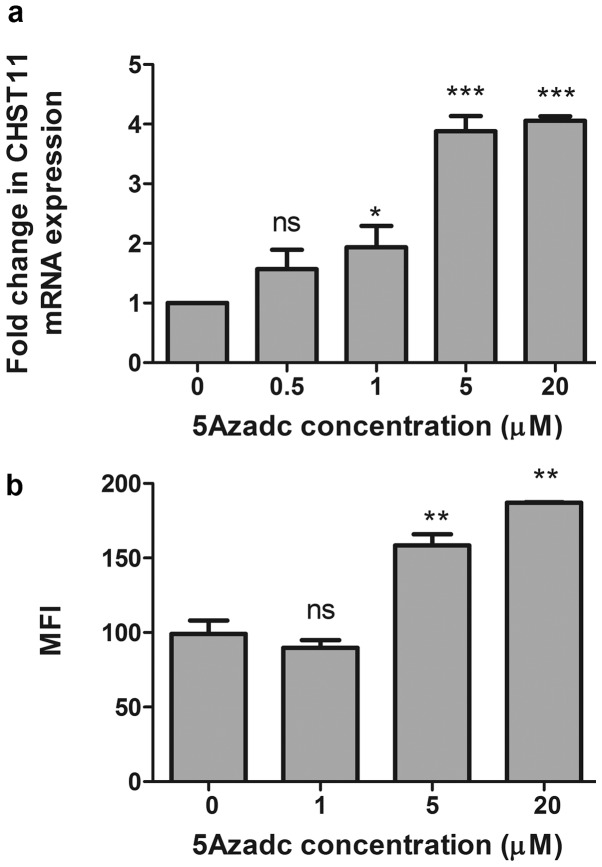
5AzadC treatment increases *CHST11* gene expression in MCF7 cells. Gene expression was assayed by real-time RT-PCR (a) and flow cytometry (b). MCF7 cells were grown in the presence of various concentrations of 5AzadC for 5 days and then cells were harvested for RNA purification or staining with anti-CS-A mAb 2H6. (a) *CHST11* mRNA levels are shown relative to mRNA levels of cells grown in medium only. GAPDH was used as the house-keeping gene to normalize mRNA-based expression data using the ΔΔCT method. Data were log transformed and subjected to one-way ANOVA with *post hoc* Tukey’s analysis. (b) Cells were harvested and then stained with anti-CS-A mAb 2H6. Binding was analyzed by flow cytometry. Mean fluorescent intensities (MFIs) of two independent experiments were log transformed and analyzed by ANOVA and *post hoc* comparison. ^*, **, ***^Significantly different compared to control at P≤0.05, P≤0.01 and P≤0.001, respectively. NS, not significant as compared to control (0 μM 5AzadC).

To further validate our data and confirm that low expression of CHST11 in the MCF7 cell line is due to hypermethylation status of the *CHST11* CpG island, MCF7 cells were treated with 5AzadC and *CHST11* gene expression was determined. Upon 5AzadC treatment, we observed a significant increase in the expression of CHST11 mRNA (Fig. 3a) that paralleled surface expression of CS-A as assayed by flow cytometry using anti-CS-A mAb 2H6 (Fig. 3b). Together, the data suggest that low expression of *CHST11* in the MCF7 cells is due to promoter hypermethylation, and that DNA hypomethylation in the more aggressive mesenchymal-like MDA-MB-231 cell line is permissive for overt *CHST11* expression.

### Methylation of the CHST11 CpG island in other cell lines with low expression of CHST11

**Figure 4:**
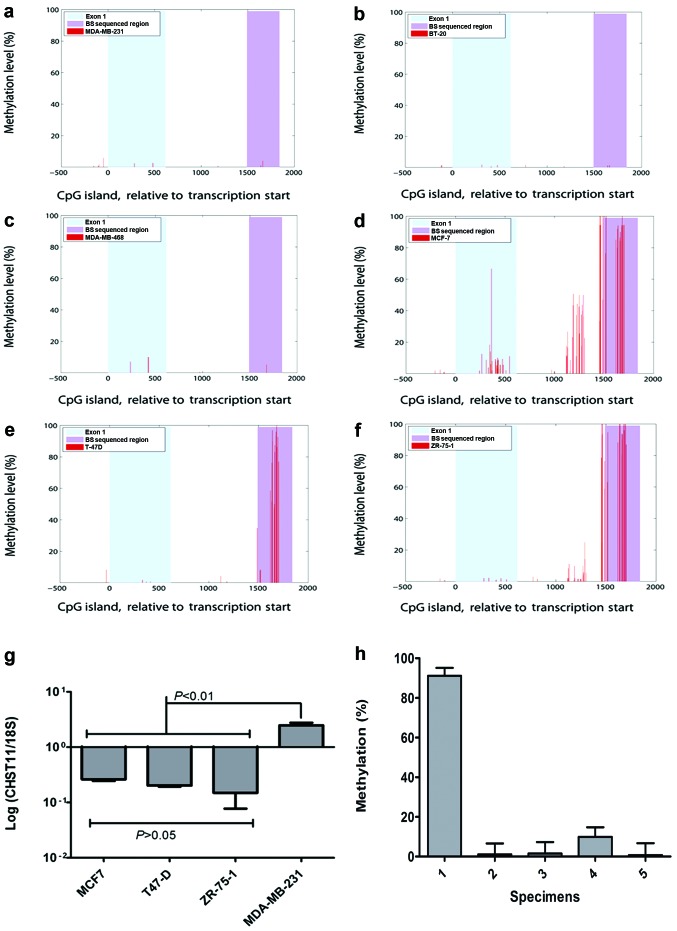
*CHST11* expression and methylation status of its CpG island in basal-like and luminal-like breast cancer cell lines and the patient tissues. Single nucleotide resolution of DNA methylation in the *CHST11* CpG island of MDA-MB-231 (a), BT-20 (b), MDA-MB-468 (c), MCF-7 (d), T-47D (e), and ZR-75-1 (f) was determined by RRBS (36). The red peaks denote Cs covered with ≥10 reads. Methylation level percentage was calculated from the number of Cs after bisulfite treatment divided by the sum of Cs (methylated sites) and Ts (unmethylated sites). There are a total of 186 CpG dinucleotides in the CpG island, of which ≥75% were accessible to RRBS with a minimum coverage of 10 (average coverage ≥40). The x-axis shows CHST11 in the coordinates relative to the transcription start site. The first exon and the genomic region we sequenced after the bisulfite treatment (BS sequenced region) are shown as indicated in the plots. (g) The expression of *CHST11* was measured in MCF7, T-47D, ZR-75-1 and MDA-MB-231 cells by qRT-PCR and normalized using 18S. Data were analyzed by one-way ANOVA and *post hoc* analysis using data from three independent experiments. Means, standard deviations, statistically significant differences and P-values are shown. (h) Methylation levels of the CpG island in five specimens from breast cancer patients diagnosed with invasive ductal carcinoma. Methylation analysis was performed and methylation levels were quantified as described in legend to Fig. 2. Percentages were averaged >20 CpGs and ≥2 replications. Means and SEM are shown. Data were Arcsin transformed and subjected to one-way ANOVA with Tukey’s *post hoc* analysis to compare means. Specimen 1 is ER-positive and the other four specimens are triple negative. Specimen 1 displayed a significantly higher methylation level than the other specimens (P≤0.001).

To confirm a role for methylation of the *CHST11* CpG island in the expression control of *CHST11*, we further analyzed second generation DNA methylation sequencing data on breast cancer cell lines (Illumina Idea Challenge and ref. 25). This method provided information on DNA methylation at single nucleotide resolution across 162 out of 186 CpGs in the ~2.5 kb long *CHST11* CpG island. Methylation analysis of Illumina Idea Challenge data of these cell lines indicates a close relationship between expression levels and methylation status. A very hypomethylated state was observed for MDA-MB-231 (Fig. 4a). The methylation status of two other triple-negative cell lines MDA-MB-468 and BT-20, was similar to MDA-MB-231 (Fig 4b and c). Similar to MCF7 cells, the CpG island of the *CHST11* gene in the T47-D and ZR-75-1 cell lines, was hypermethylated (Fig. 4d–f). The expression of *CHST11* in T47-D and ZR-75-1 cell lines was further examined by qRT-PCR (Fig. 4g) and flow cytometry (Table III), and compared with *CHST11* expression levels in MCF7 and MDA-MB-231. Consistent with the methylation data, the expression of the *CHST11* gene and CS-A in both of these cell lines was significantly lower than in MDA-MB-231 and comparable with that of MCF7 cells. MDA-MB-468 displayed a very hypomethylated state but the expression of *CHST11* in these cells was moderate and less than what we observed for MDA-MB-231 and MDA-MET (4,14). The expression of *CHST11* in tumor tissue may also be controlled by DNA methylation similar to that observed in cell lines.

To determine whether the same CpG island can be methylated differently in actual breast cancer, we examined the methylation status of the CpG island of *CHST11* gene in 5 de-identified clinical breast cancer specimens. We observed that in 4 triple negative (TN, i.e., negative for ER, Her2/neu and progesterone receptor) specimens the CpG island was highly hypomethylated, very similar to what was observed for ER-negative basal-like cell lines (Fig. 4h). The CpG island was hypermethylated in one ER-positive specimen tested. Therefore, methylation status of *CHST11* CpG might be a useful marker to differentiate between luminal and basal-like breast cancer subtypes, however, a larger sample size is required to confirm this.

## Discussion

We have previously shown that removal or blocking of CS on the surface of breast cancer cells inhibits metastasis (5,14). We have demonstrated that the expression levels of *CHST11* correlate with the expression of P-selectin-reactive surface CS and aggressiveness of human breast cancer cells (14). Here, we confirm that the *CHST11* gene is overexpressed in cancer tissues compared to normal breast tissues and its expression correlates with more aggressive basal-like and HER2-amplified phenotypes in cell lines. The expression analysis of *CHST11* in DCIS and IDC specimens suggest a progression specific role for this gene that needs to be further validated. An association between *CHST11* expression and distant metastasis in breast cancer patients has been reported (6). The data suggest that the expression of *CHST11* correlates well with properties associated with prognosis and progression and should be investigated as a potential biomarker.

Our data suggest that the expression of *CHST11* is controlled by DNA methylation. Aberrant DNA methylation is involved in tumor initiation and progression (22,23,38). Hypermethylation of tumor suppressor genes is a common mechanism of gene silencing observed in cancer, and similarly, DNA hypomethylation can contribute to overexpression of tumor-promoting genes. DNA hypomethylation is associated with advanced stages, metastatic phenotypes, and drug-resistant variants of breast cancer (39–41). We find that induced hypomethylation by 5Azadc led to *CHST11* overexpression and increased levels of the *CHST11*’s immediate cell surface product CS-A. Based upon our results, it seems that a combination of hypomethylation and high expression occurs in basal-like cancer cells. Hypermethylation of *CHST11* (and very low to no expression) clearly differentiates the least aggressive, luminal cells with epithelial morphology from the more aggressive cells we tested. We observed a hypomethylated CpG island in a limited number of triple negative specimens from patients. Triple-negative cancers are usually classified as basal-like while ER-positive cells are considered luminal. The data suggest that methylation analysis of this CpG island of *CHST11* might be potentially used as a surrogate for detection of expression levels of this gene in clinical samples. However, more experiments are needed to evaluate the correlation of the expression of this gene with its DNA methylation levels among subtypes of breast cancer.

Our study illustrates an example of a gene, where methylation is lower and expression is higher as the cancer phenotype becomes more aggressive. In contrast, the main trend is widespread gene hypermethylation as cancers go from less aggressive to more aggressive phenotypes (42,43). Our data are consistent with some other models of metastatic and/or more aggressive cancers exemplifying tumor promoting genes like urokinase plasminogen activator (uPA), PAX3 and Ezrin that are less methylated and/or more highly expressed in the more aggressive cancer forms (40,44,45). This less frequent pattern of gene methylation between cancers with low and high aggressiveness suggests that the expression of these genes is necessary for the more aggressive phenotypes (because their change in gene specific methylation is counter to the trend of gene hypermethylation in cancer).

It needs to be pointed out that in less aggressive basal B cells that are epithelial-like (e.g., MDA-MB-468), hypomethylation is accompanied by intermediate expression of *CHST11*, suggesting involvement of other mechanisms in controlling the expression levels of this gene such as TGFβ1 (46,47). As we suggested before (14), in order for CHST11 activity to express the proper receptor it might need to be co-expressed with a specific proteoglycan. Future studies on well-characterized clinical specimens are needed to determine a role for the *CHST11* gene alone or in combination with other genes in patient outcome. More studies are also needed to determine whether DNA methylation status can replace gene expression for prognosis.

The recognition that silencing of tumor suppressor genes through promoter hypermethylation plays a significant role in tumorigenesis (48,49) has led to the clinical use of hypomethylating agents including 5AzadC (50). However, the expression of several pro-tumor genes is induced by DNA hypomethylation (40,45). The expression of such genes, including *CHST11*, may be activated by the clinical use of hypomethylating agents and this may promote more aggressive forms of breast cancer. In this regard, our data, in agreement with others (51), suggest that therapeutic use of such demethylating agents may promote tumor progression and metastasis. Histone deacetylase inhibitors can also hypomethylate genes and change their expression levels in breast cancer cell lines (52) and their combination with metabolic therapies may modify their action (53). Therefore, additional studies are needed to determine if some agents or mechanisms of hypomethylation are more or less likely to promote tumor metastasis.

Our findings suggest that the expression of *CHST11* correlates with aggressive phenotypes and progression of DCIS to IDC. We found out that the expression of *CHST11* is modulated by DNA methylation. Therefore, DNA methylation plays an important role in the remodeling of CS in breast tumors. Our data strongly suggest that the expression of *CHST11* and its role in defining metastatic potential of tumor cells should be seriously considered when demethylating agents are used for medical treatment of breast cancer patients. Moreover, the methylation and expression of the *CHST11* gene have potential to be developed into novel prognostic biomarkers.

## Figures and Tables

**Table I:** Primers used in this study.

Primer	Sequence
18S forward	5′-TTCGAACGTCTGCCCTATCAA-3′
18S reverse	5′-ATGGTAGGCACGGCGACTA-3′
*CHST11* forward	5′-TCCCTTTGGTGTGGACATCT-3′
*CHST11* reverse	5′-CACGTGTCTGTCACCTGGTC-3′
GAPDH forward	5′-ACAGTCAGCCGCATCTTCTT-3′
GAPDH reverse	5′-ACGACCAAATCCGTTGACTC-3′
*CHST11* bisulfite outside, forward	5′-TTTGATTATTGTAGTTTTGGAGGAAAT-3′
*CHST11* bisulfite reverse	5′-CCTTACAATTAAAAAAACAAATTATTACTA-3′
*CHST11* bisulfite nested, forward	5′-TTTTTGTGGTTTAGGTAAAGTTTTA-3′

18S, 18S ribosomal RNA; *CHST11*, carbohydrate (chondroitin 4) sulfotransferase 11; GAPDH, glyceraldehyde-3-phosphate dehydrogenase.

**Table II:** *CHST11* differential transcript expression in human breast carcinomas extracted from multiple studies in the Oncomine microarray database.

Study^a^	Comparison (specimen number in each group)	Average fold increase	P-value^b^
TCGA^c^	Invasive breast carcinoma (n=76) vs normal (n=61)	2.6	2.51E-24
Finak *et al* (27)	Invasive breast carcinoma (n=53) vs normal (n=6)	3.6	9.43E-20
Richardson *et al* (26)	Ductal breast carcinoma (n=40) vs normal (n=7)	2.2	2.00E-4^d^
Gluck *et al* (28)	Invasive breast carcinoma (n=154) vs. normal (n=4)	2	0.02

a Studies are addressed by consortium name or first author’s last name.

b Two-tailed P-values are shown.

c TCGA, The Cancer Genome Atlas - Invasive Breast Carcinoma Gene Expression Data (http://tcga-data.nci.nih.gov/tcga/).

d Student’s t-test was performed with Welch’s correction for unequal variances.

**Table III:** Summary of *CHST11* gene expression and methylation status in human breast cancer cell lines tested.

Cell line	ER status	Molecular subtype	CHST11 (qRT-PCR)	CS-A expression (2H6 mAb binding by flow cytometry)	DNA methylation status
MCF7	Positive	Luminal	Low	Low^b^	Hypermethylated
T47-D	Positive	Luminal	Low	Low^c^	Hypermethylated
ZR-75-1	Positive	Luminal	Low	Low^c^	Hypermethylated
BT-474	Positive	Her-2 amplified	Low^a^	ND	Hypermethylated
MDA-MB-468	Negative	Basal-like	Intermediate	Intermediate to high^b^	Hypomethylated
BT-20	Negative	Basal-like	Intermediate^a^	ND	Hypomethylated
MDA-MB-231	Negative	Basal-like	High	High^b^	Hypomethylated
MDA-MET	Negative	Basal-like	High	High^b^	Hypomethylated

a Digital gene expression with primers used to perform qRT-PCR was applied to estimate gene expression. ND, not determined.

b Flow cytometry data were published before (14).

c Flow cytometry was performed and no detectable binding of anti-CS-A mAb 2H6 was observed.
